# Real-time tracking of protein unfolding with time-resolved x-ray solution scattering

**DOI:** 10.1063/4.0000013

**Published:** 2020-09-22

**Authors:** L. Henry, M. R. Panman, L. Isaksson, E. Claesson, I. Kosheleva, R. Henning, S. Westenhoff, O. Berntsson

**Affiliations:** 1 Department of Chemistry and Molecular Biology, University of Gothenburg, 40530 Gothenburg, Sweden; 2 Center for Advanced Radiation Sources, The University of Chicago, Chicago, Illinois 60637, USA; 3 MAX IV Laboratory, Lund University, Lund 221 00, Sweden

## Abstract

The correct folding of proteins is of paramount importance for their function, and protein misfolding is believed to be the primary cause of a wide range of diseases. Protein folding has been investigated with time-averaged methods and time-resolved spectroscopy, but observing the structural dynamics of the unfolding process in real-time is challenging. Here, we demonstrate an approach to directly reveal the structural changes in the unfolding reaction. We use nano- to millisecond time-resolved x-ray solution scattering to probe the unfolding of apomyoglobin. The unfolding reaction was triggered using a temperature jump, which was induced by a nanosecond laser pulse. We demonstrate a new strategy to interpret time-resolved x-ray solution scattering data, which evaluates ensembles of structures obtained from molecular dynamics simulations. We find that apomyoglobin passes three states when unfolding, which we characterize as native, molten globule, and unfolded. The molten globule dominates the population under the conditions investigated herein, whereas native and unfolded structures primarily contribute before the laser jump and 30 *μ*s after it, respectively. The molten globule retains much of the native structure but shows a dynamic pattern of inter-residue contacts. Our study demonstrates a new strategy to directly observe structural changes over the cause of the unfolding reaction, providing time- and spatially resolved atomic details of the folding mechanism of globular proteins.

## INTRODUCTION

Correctly folded proteins are one of the cornerstones of life. Tremendous advances have been made in the protein folding field in the last 60 years to understand the folding mechanism of proteins.^1^ One example is the hydrophobic-collapse mechanism. In this concept, hydrophobic residues collide in the early events of protein folding into a localized secondary structure, forming a folding nucleus. The rest of the protein folds rapidly onto the hydrophobic nucleus, enabling the acquisition of the native packing interactions.^2^

Protein unfolding is involved in many natural cellular processes such as membrane translocation or adenosine triphosphate (ATP)-dependent protein degradation.^3^ In recent years, it has been discovered that protein misfolding, unfolding, and aggregation are also associated with pandemic amyloid diseases^4^ and cancer.^5,6^ Therefore, understanding the mechanism of protein unfolding has become imperative.

**FIG. 1.**
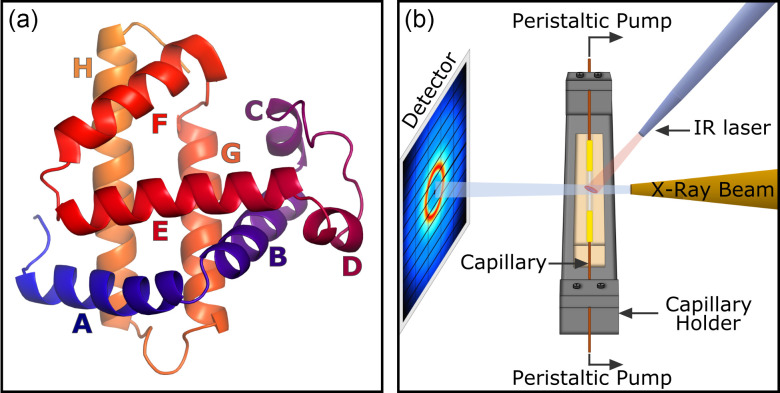
ApoMb and experimental setup. ApoMb model obtained after the removal of biliverdin from the published apomyoglobin structure (PDB accession code: 1BVC) is presented in (a). The protein is composed of eight *α*-helices A: blue to H: orange, where helices A, G, and H form the hydrophobic core. The experimental setup is schematized in (b).

In order to advance the field, the study of folding processes on relevant timescales and with a structural probe is needed. Apomyoglobin (apoMb) is a representative of a group of relatively small, *α*-helical and globular proteins [Fig. 1(a)]. The folding pathway of apoMb has been intensely studied using nuclear magnetic resonance (NMR),^7–12^ and circular dichroism (CD).^13,14^ It is postulated that apoMb unfolding can be described by a three-state model, like many other proteins of similar size: from the native state to an intermediate state to an unfolded state. The intermediate state, stabilized and trapped at low pH, has been identified as a molten globule. It retains slightly more than half of the *α*-helices of the native state.^13^ Described by small-angle x-ray scattering (SAXS), this intermediate has a radius of gyration of 23 Å compared to 19 Å for the native state and 34 Å for the unfolded state.^15^ The non-polar core of the traditionally observed molten globule has been found to be hydrated.^16^ The acid-unfolded molten globule has also been characterized on the atomic level by NMR and maintains a hydrophobic core formed by helices A, G, and H, the G–H loop,^10^ and part of helix B.^17^ It is known that the A, G, and H helices, as well as the carboxy-terminal part of helix B, associate in the early stages of apoMb folding. However, these findings arise from the (equilibrium) acid-unfolded state. The presence and transition between those states have not yet been observed in real-time. A structural probe, which follows the time-course of the unfolding reaction, is needed.

Several techniques have been used to study protein folding, such as NMR, CD, hydrogen exchange (HX) pulse labeling, spectroscopy, and Fluorescence Resonance Energy Transfer (FRET).^18–24^ Many of these techniques, like spectroscopy, are only indirectly informing on the structure of the protein,^22,23,25^ and some, such as FRET and CD methods, are only sensitive to the local environment surrounding a specific residue.^18,21,26,27^ In addition, many of these studies encounter difficulties to reach the timescales of the early folding events, achieved in less than few seconds, and rather focus on studying protein folding under equilibrium conditions (NMR^11,28,29^ and HX pulse labeling^30,31^). X-ray solution scattering is sensitive to the distance between the electrons that scatter the x-rays.^32^ Thus, the technique directly probes the structure of the protein in solution. It also explores structures on multiple length scales, ranging from interactions between protein molecules, through the global shape, to the tertiary and secondary structures of the protein.^33–36^ SAXS has been used to study unfolding under equilibrium conditions^37^ as well as on millisecond timescales following rapid mixing.^15^ By combining x-ray solution scattering with a laser pulse that triggers a conformational change in the sample, it is possible to follow structural changes over an even wider time range. Difference time-resolved x-ray solution scattering (TRXSS) can then directly detect changes in distances between electrons after a laser pulse perturbation.

Since TRXSS focuses on what is different between two measurements, it is able to detect more subtle changes in structure, compared to conventional SAXS.^34,38–41^ However, extracting the structural information from the solution scattering data is a major challenge.^42^ With no or minimal modeling, it is possible to derive information such as the radius of gyration (*R_g_*), the maximum dimension (*D_max_*), the average envelope, and the shape of the particle of interest.^43^ More detailed analysis requires the scattering to be predicted from atomic models, for which there are several tools available.^44–47^ In many cases, however, it may not be correct to assume that only one relatively well-defined model represents the protein structure in solution. On the contrary, one can expect to find a mixture of structurally different substrates in equilibrium. Therefore, instead of considering only one structure and performing a classical kinetic modeling as usually done in analyzing TRXSS data analysis,^34,38–40,48^ ensembles of several structures should be considered. Such an approach is available for SAXS analysis,^49^ but not for TRXSS, where difference scattering is analyzed. Here, we present a method to refine ensembles for TRXSS, where the data are structurally interpreted in terms of ensembles of structures, generated by Molecular Dynamics (MD) simulations, and selected via a genetic algorithm. With this approach, we study the unfolding of apoMb on a nano- to millisecond timescale following a laser induced temperature jump (T-jump).

## MATERIALS AND METHODS

### Sample preparation

Myoglobin from equine skeletal muscle was purchased from Sigma-Aldrich^®^. ApoMb was prepared by removing the heme according to the acid methyl ethyl ketone (MEK) method.^50^ Myoglobin (0.4 g) was dissolved in 20 ml MilliQ-water and the pH was adjusted to 1.87 with HCl. Ice cold MEK (20 ml) was added and the solution was shaken. The low pH caused myoglobin to unfold and the heme to separate into the organic phase. The organic phase was removed and another 20 ml MEK was added. This procedure was repeated two more times. In order to refold the protein, the solution was then dialyzed overnight (6–8 kDa cutoff) against a solution of sodium acetate (10 mM, pH 5.5). Some flocculates were formed and removed by centrifugation, 2 × 10 min at 4000 rcf and 4 °C. The sample was concentrated to 25 mg/ml, using 10 kDa MWCO Vivaspin concentration tubes (Sartorius^®^). By comparing the absorbance of holo- and apoMb (409 vs 280 nm), the solution was judged to consist of more than 99% apoMb. The samples were divided into 0.5 ml aliquots, frozen in liquid nitrogen, and stored at −80 °C until further use. Prior to any experiment, the samples were filtered through a 0.2 *μ*m spin-filter (VWR^®^).

### Thermal unfolding assay

To perform the unfolding assay, 2 ml of apoMb (25 mg/ml) were thawed and filtered through a 0.2 *μ*m centrifugal-filter (VWR). Triplicate measurements were run on Prometheus NT.48 (NanoTemper^®^) from 35 °C to 98 °C. The ratio of tryptophan emission at 330 nm and 350 nm, which describes the shift of tryptophan emission upon unfolding, was measured. The melting temperature *T_m_* (56 °C), at which half of the protein population is unfolded, was extracted from the curve. Based on these results, and in accordance with previous studies,^51^ the ground temperature in the x-ray scattering measurements was set to 50 °C.

### X-ray solution scattering data collection and processing

X-ray solution scattering data were acquired at the BioCARS beamline (ID14-B) at the Advanced Photon Source (APS). The pink beam^52^ with a nominal energy of 12 keV and a bandwidth of ca. 3% was used, and the x-rays were focused to a 35 × 38 *μ*m^2^ spot at the sample position. The x-ray flux was ca. 7.3 *μ*J per single pulse (equal to ca. 1.7 mJ/mm^2^). The sample was continuously pumped by a peristaltic pump (0.5–2 *μ*l/s) and circulated in a 0.7 mm diameter quartz capillary [Fig. 1(b)]. The capillary was fixed on a temperature-controlled (50 °C), aluminum nitride capillary holder as described by Rimmerman *et al.*^53^

Conventional SAXS data were collected at 50 °C at protein concentrations of 1.7, 3.3, 6.4, and 12.7 mg/ml. The center of the x-ray beam was placed at the center of the capillary. Ten images were taken at each concentration, as well as ten images of buffer solution prior to and after each protein measurement, using the 11/24 bunch mode and 40 pulses per image. The data were averaged and subtracted using the ATSAS package.^54^ The final steady-state data were obtained by merging the averaged data of 3.3 and 12.7 mg/ml using an overlap between 0.09 < *q* < 0.14 Å^−1^, where *q* is the scattering vector (q=4π(sin (θ))/λ) with 2*θ* being the scattering angle and *λ* the x-ray wavelength. The pair distance distribution function was computed using GNOM (v 5.0) over 0.035 < *q* < 0.44 Å^−1^.

Time-resolved x-ray scattering data were collected at 12.7 mg/ml and recorded following a T-jump. The T-jump was triggered by a 7 ns IR laser pulse (1443 nm) with an energy density of ca. 49 mJ/mm^2^, focused on a spot aligned with the x-rays on the capillary [Fig. 1(b)]. The x-ray and laser are placed at 90° to each other and the center of the x-ray beam is ca. 250 *μ*m from the top of the capillary where the laser is focused. The size of the laser spot was adjusted so that it engulfed the x-ray spot. T-jump and x-ray irradiation were repeated with a frequency of 20 Hz. The increase in temperature was determined by comparing the water scattering after the T-jump to the difference of the static water scattering at different temperatures ranging from 25 to 40 °C. The magnitude of the T-jump was determined to be 10 ± 1 °C and is in accordance with previous measurements.^53^ The temperature increase will not be homogeneous throughout the solvent, but the volume probed by the x-ray will display some thermal gradient both across and along the x-ray beam, and the measured T-jump is the average T-jump observed by the x-rays. The thermal gradient relevant to this experiment has been simulated (see supplementary material for further details, Fig. S1). The temporal offset between the laser and x-rays was adjusted to collect data ranging from 10 ns to 20 ms after the T-jump. Interleaved with these time points, we also collected data where the x-ray pulse arrives 5 *μ*s before the laser pulse; this then represents the unperturbed reference signal. Depending on the timescale investigated, 24/24 or 1/24 bunches were used and the number of pulses per image was adjusted to keep the total number of x-ray pulses collected for each image relatively constant. Detector images were radially integrated using our own software and normalized to the average scattering in the region 2 < *q* < 2.2 Å^−1^, where water displays an isosbestic point with respect to heating,^55^ and the protein contribution to the scattering curve is small. Absolute scattering data, deviating from the median by more than two standard deviations in the region 2 < *q* < 2.5 Å^−1^, were rejected. Difference data were obtained by subtracting the reference signal recorded 5 *μ*s before the IR laser trigger. The outlier-rejection for the difference scattering data was performed for each time-point with a cutoff of one standard deviation for 0.06, 0.1, and 0.3 *μ*s, two standard deviations for 0.01, 0.02, 0.6, 1, 2, and 3 *μ*s, and finally three standard deviations for 0.03, 0.2, and 10–600 *μ*s. Typically, about 85% of the raw data were considered for further analysis. Subsequently, the scattering curves corresponding to the same x-ray-laser time-delay were merged and averaged together.

To retrieve scattering curves representing only the protein structural change, the contributions of solvent heating and varying capillary thickness had to be removed. To do so, the scattering of the heated solvent alone was recorded at different depths into the capillary from the point where the laser meets the capillary (resulting in a slightly larger or smaller fraction of capillary in each image) and at 10 ns and 100 *μ*s delay times. At 10 ns and 100 *μ*s, the difference scattering is mostly, but not totally, due to density changes at constant volume and at constant pressure, respectively.^56^ The data for the two time points, and different depths, were subjected to an singular value decomposition (SVD), yielding three components above noise. The three components were scaled to the data, in the range 1.5 < *q* < 2.95 Å^−1^ where water scattering dominates, and finally subtracted (Fig. S2). A drop of sample temperature, due to thermal diffusion, was observed from 1 ms. We, therefore, only treated the delay times up to 600 *μ*s to study the thermal unfolding process of apoMb.

**FIG. 2.**
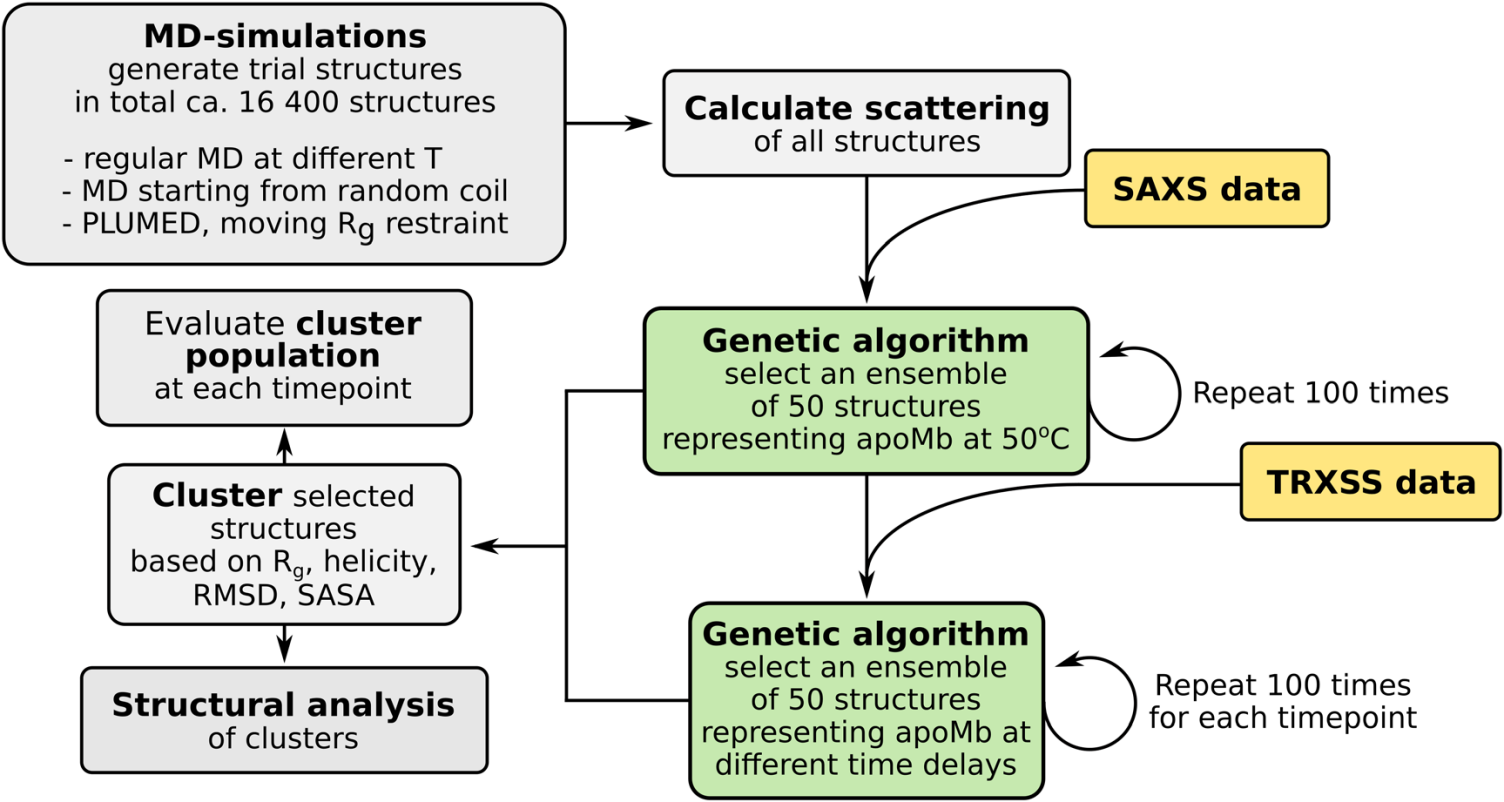
A block diagram showing the data analysis strategy employed.

Prior to any structural analysis, the structure factor contribution was removed from the experimental difference scattering data. The (difference) scattering signal depends on the shape of the solute [form factor, *P*(*q*)] and the interactions between different solute particles [structure factor, *S*(*q*)] as *I*(*q*) ∝ *P*(*q*)*S*(*q*). From the conventional SAXS measurement, *S*(*q*) is obtained by dividing the high concentration scattering curve with a low concentration scattering curve. The structure factor is only truly different from one at low angles. High angle differences caused by errors in background subtraction, etc., are disregarded, and *S*(*q* > 0.15 Å^−1^) is set to 1. This removal of *S*(*q*) relies on the assumption that *S*(*q*) does not change with increasing temperature on the time scales investigated here. According to Gilmanshin *et al.*,^51^ aggregation is expected only on longer timescales, validating the described procedure. An alternative approach, which does not rely on this assumption, would be to record TRXSS data at different concentrations. However, restraints in the form of available beamtime and/or sample availability often make this impractical.

### Molecular dynamics simulations

To generate sensible trial models for the structural analysis of the TRXSS data, atomic models were generated via classical Molecular Dynamics (MD) simulations using GROMACS 5.0.4^57^ and the CHARMM27 force field.^58^ To maximize the conformational space sampled by the simulations, eleven simulations, of 100 ns each, were performed, covering every 20 K, from 300 K to 520 K. The crystal structure of apoMb (PDB accession code: 1BVC^59^) after the removal of the biliverdin ligand was used as the initial model.

The protein was placed in a cubic box, 1 nm larger than the protein in all directions. The system was solvated with transferable inter-particle potential with three points (TIP3P) water molecules and replicated in all directions with periodic boundary conditions. The protonation state of the different residues was chosen as to represent the protonation of apoMb at pH 5.5, as predicted by PROPKA.^60^ The system was neutralized by replacing water molecules with sodium and chloride ions to reach a final concentration of 150 mM NaCl. After adding ions, the system underwent initial energy minimization until all forces were below 1000 kJ/mol/nm^2^. Subsequently, the system was minimized for 100 ps in the canonical (NVT) and isothermal–isobaric (NPT) ensembles. During equilibration, all non-hydrogen atoms were position restrained with force constants of 1000 kJ/mol/nm^2^. All bonds were constrained using the linear constraint solver algorithm, and a time step of 2 fs was used. Particle mesh Ewald (PME) electrostatics with fourth-order interpolation and with a grid spacing of 0.12 nm was used. The cutoff scheme was Verlet with a 1.0 nm cutoff, and cutoffs for short-range electrostatic and van der Waals interactions were 1.0 nm as well. During the 100 ns production run, pressure control was achieved using the Parrinello–Rahman barostat (*τ_P_* = 2 ps, P = 1 bar), and temperature control was achieved via the modified Berendsen (velocity-rescale) thermostat (*τ_T_* = 0.1 ps; T = 300 K). From these simulations, we obtained 12 012 structures.

Two additional sets of MD simulations were performed to further increase the sampled conformational space. One of these sets was starting from a random coil structural model of apoMb generated with the Bax Group's Extended structure server.^61^ This simulation was performed as just described, at 300 K and ca. 40 ns, resulting in 402 structures. In addition, we performed simulations to restrain the radius of gyration (*R_g_*) using PLUMED,^62,63^ to account for the structural gap, in *R_g_*, solvent accessible surface area (SASA), and root mean square deviation (RMSD) with respect to the crystal structure (Fig. S3) between the standard and the random coil simulations. PLUMED simulations were run at 420 K and started from the same crystal structure as previously described for the temperature unfolding simulations. The moving restraint was set to increase the *R_g_* from 15.3 Å to 32 Å over 5 000 000 steps, by applying a *κ* of 100 000. The moving restraint simulations generated 4004 structures.

**FIG. 3.**
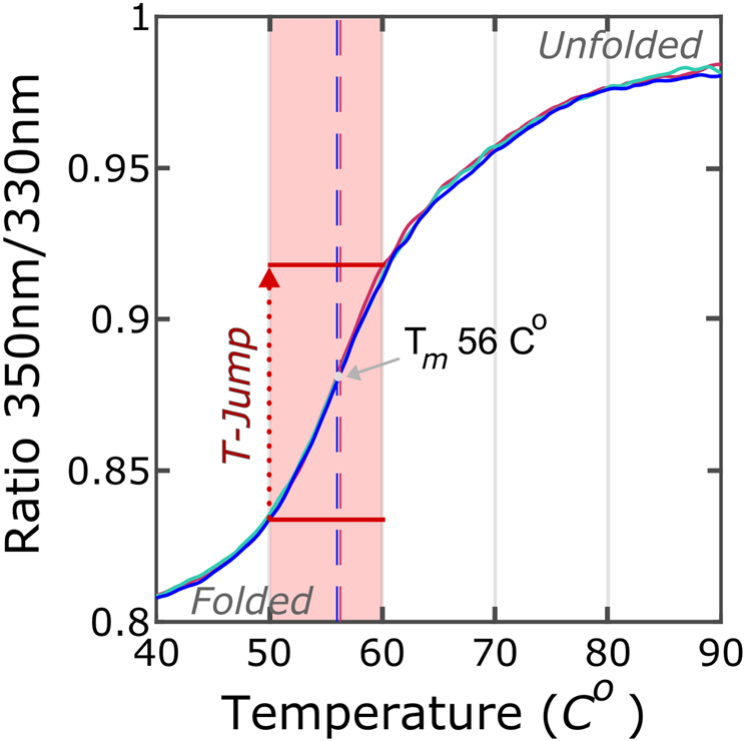
Thermal unfolding of apoMb by tryptophan fluorescence. The unfolding transition midpoints (dashed line), for each replicate (three in total), were determined automatically from the first derivative of the fluorescence ratio (F350/F330). The T-jump, from 50 to 60 °C (area shaded in red), is represented by red dots.

### TRXSS filtered MD simulations and structural modeling of the data

We chose not to fit single structures to the data but ensembles of structures. The data analysis strategy is displayed as a block diagram in Fig. 2. Ensembles of structures that reproduce the steady-state and difference data were selected using a genetic algorithm. The genetic algorithm is very similar to that of the ensemble optimization method (EOM) in the ATSAS suite of analysis tools,^49^ but was adapted to fit difference scattering data.

The scattering of atomic models, generated by MD simulations, was predicted using CRYSOL.^44^ There are several methods available for predicting the scattering from atomic coordinates. A notable example is WAXSiS,^46^ which uses an explicit solvent model to predict the scattering of the hydration layer surrounding the protein. While this method is most likely somewhat more accurate, particularly at high *q*,^64^ it is also currently too computationally intensive for the application described in this contribution. Another benefit of CRYSOL is the ease to vary the contrast of the hydration layer, which can be expected to occur during protein (un)folding. Therefore, we decided to use CRYSOL to predict the scattering of the pre-generated atomic models. CRYSOL was used with the number of spherical harmonics set to 50, the order of the Fibonacci grid to 18, and explicitly accounting for hydrogen atoms. The scattering was predicted on a *q*-grid with a spacing of 0.0025 Å^−1^. For each structure, six scattering curves were calculated varying the contrast of the hydration layer from 0 to 0.05 e/Å^3^ in steps of 0.01 e/Å^3^. As mentioned, the pink beam was used. This means that there is a distribution of energies in the x-ray beam. This causes smearing of the recorded data and has to be accounted for. The predicted scattering was convoluted with the pink beam wavelength distribution following Ref. 65.

Together with the predicted scattering, the experimental data provide input to the genetic algorithm. From the pool of predicted scattering (from 16418 structures at six different hydration layer contrasts, gene pool) 50 ensembles (chromosomes), each containing 50 scattering curves (genes), were randomly selected. Over successive generations, the chromosomes evolved toward the optimal solution, which would fit our experimental data. In this way, the SAXS and TRXSS data were used to provide global restraints for the analysis and selection of atomic models. During each generation, the chromosomes were subjected to random mutation and crossing. Random mutations allowed up to 20% of the genes of a chromosome to be exchanged for others, where 50% of these came from the other chromosomes of the same generation and 50% came from the gene pool. During the crossing operation, genes from two chromosome selected randomly were exchanged. Following the mutation and crossing, there were 150 chromosomes used for fitting. The average scattering of each of these chromosomes was evaluated against the experimental data (allowing the scale and offset to be adjusted). The ensemble refinement was performed in iterations. For every repeat, the 50 best scoring chromosomes were selected for further evolution. In total, 100 repeats were performed, for each time point. For each repeat, the stopping criteria were set to 2000 generations or when less than 1% fit improvement was observed after 300 consecutive generations. On average, the genetic algorithm converged after ca. 900 generations.

Using a *q*-range from 0.03 to 0.3 Å^−1^, the experimental data were used to provide restraints on the overall structure. We chose this *q*-range because even course methods such as CRYSOL are expected to be fairly accurate throughout this range. For the steady-state SAXS, the genetic algorithm was run directly against the steady-state scattering data. For the difference scattering data, the scattering of the average best ground state ensemble was subtracted from each ensemble before running a genetic algorithm. The scale and offset were allowed to vary by 30% from the ground state scaling factors. This is because initially, the TRXSS data and SAXS data are not on the same scale, and there is a certain degree of uncertainty in finding the scale factor between these two. This process resulted in 100 ensembles, each with 50 structures, for each scattering curve (SAXS and TRXSS). Not all repeats converged to an acceptable agreement between experimental and modeled data, and the best scoring quartile was further analyzed. The convergence of the genetic algorithm over the 25 best repeats was verified by assessing the distribution and average *R_g_*, SASA, helicity, and RMSD with respect to the crystal structure of each ensemble at each time delay.

Finally, the structures selected by the genetic algorithm were sorted in an agglomerative hierarchical cluster tree. The atomic models, obtained by filtered MD simulations, were grouped according to their normalized *R_g_*, SASA, RMSD, and helicity using the Ward “minimum variance” cluster algorithm and Euclidean distances (using MATLAB's linkage and cluster function). At each step of the clustering algorithm, two clusters are merged. The pairs of clusters, which result in the smallest variance of the merged cluster, are selected. Using a cutoff of 6 normalized distance units, three clusters were identified.

### Structural characterization of the atomic models

The tools in GROMACS 5.0.4 were used for the structural analysis of the atomic models obtained by TRXSS-filtered MD simulations using a genetic algorithm. For each structure, we calculated the *R_g_*, the RMSD of each atomic model with respect to the crystal structure, and the protein SASA. The software DSSP^66^ was used to predict secondary structures for each cluster model. Contact maps were calculated for all the selected models by finding distances between residue pairs smaller than 0.4 nm.

## RESULTS

### Tracking the unfolding of apoMb using T-jump TRXSS

**FIG. 4.**
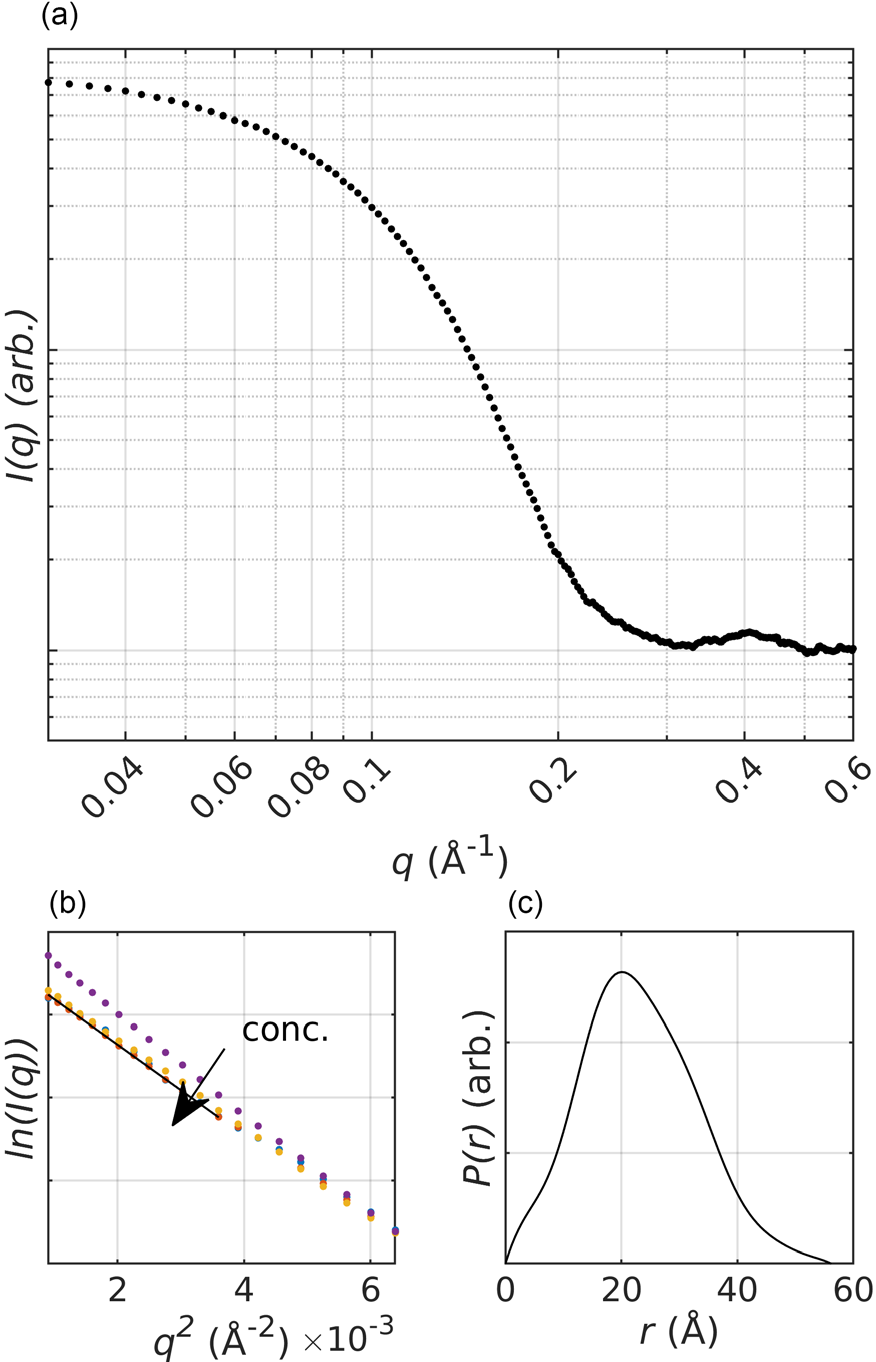
Steady-state SAXS data for apoMb at 50 °C (a). The Guinier analysis (b) of the dilution series shows that the concentration dependent structure factor caused by inter-particle interactions is essentially 1 for *q* >0.07 Å^−1^ (*q*^2^ >0.005 Å^−2^). The pair distribution function of the data is typical for a globular protein (c).

**FIG. 5.**
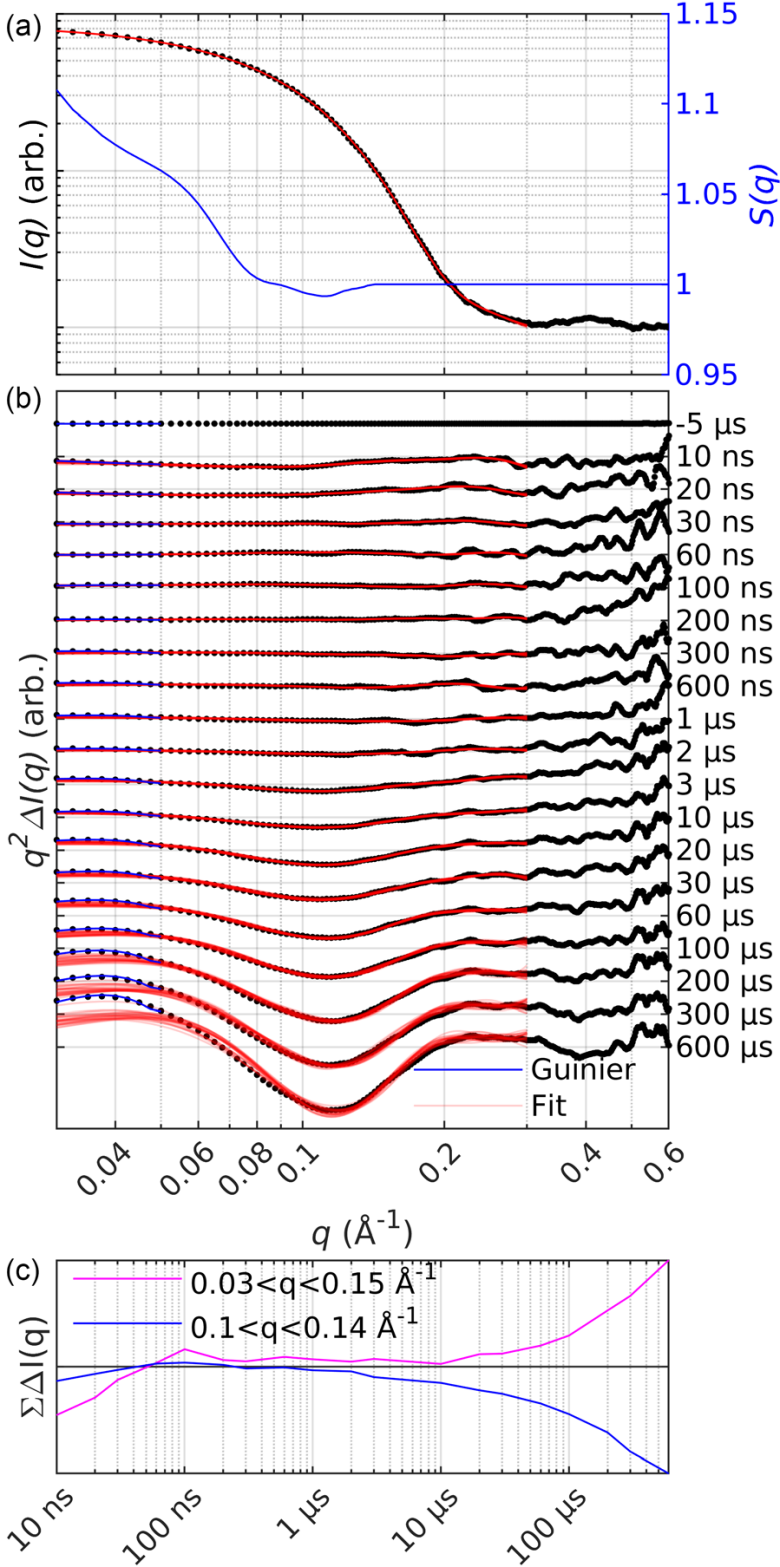
Steady-state SAXS data at 50 °C (black dotted) and structure factor (blue) (a). Predicted scattering from the 25 best scoring ensembles is shown as red curves. Panel (b) shows the TRXSS data following a laser induced T-jump in gray dots. The predicted scattering from the 25 best scoring ensembles at each time point is shown as red curves and the Guinier fit at low *q* as a blue curve. Summed intensity in for 0.03 < *q* < 0.15 Å^−1^ (magenta) and 0.1 < *q* < 0.14 Å^−1^ (black) (c).

Using tryptophan fluorescence (Fig. 3), the melting temperature ($T_{m}$) of apoMb in 10 mM sodium acetate (pH 5.5) was measured to be 56 °C. This is similar to what was reported in Ref. 51, but a bit lower than what was reported in, for example, Ref. 67. The $T_{m}$ values are sensitive to the salt concentration.^67^ In contrast to Huang *et al.*^67^ where the experiments were performed in the absence of salt, the experiments described herein were performed in 10 mM sodium acetate, and Gilmanshin *et al.*^51^ performed their experiments in 10 mM NaCl. This may account for the difference in measured $T_{m}$. Based on the observed melting temperature, we performed SAXS measurements of apoMb at 50 °C [Figs. 4 and 5(a)]. Analysis of the SAXS data yielded a *R_g_* (ca. 18 Å) and a maximum particle dimension (*D_max_*, ca. 56 Å) [Table I, Figs. 4(b) and 4(c)], which are in agreement with the values previously reported for the folded apoMb.^37,68^

**TABLE I.** Sample details, data collection parameters, SAXS structural parameters, data processing, and structural modeling information.

Sample details	
Sample name	Apomyoglobin
Origin	Horse skeletal muscle
Source (catalog number)	Sigma-Aldrich (M0630)
Additional preparation	Yes, see “Materials and Methods”
Buffer	10 mM Na-acetate, pH 5.5
Data collection parameters	
Beamline	BioCARS, 14ID-B (APS)
X-ray energy (keV)	12 (pink beam)
Bandwidth (%)	ca. 3
*q* Definition	*q* = 4 π sin (θ)/λ
	Angle 2*θ*, x-ray wavelength *λ*
X-ray spot (*μ*m × *μ*m)	35 × 38
Protein concentration (mg/ml)	1.7–12.7,^a^ 12.7^b^
Temperature (°C)	50
X-ray exposure per image	40 × 11/24 bunches^a^
	100 × 24/24, 2000 × 1/24^b^
Laser pulse duration (ns)^b^	7
Laser wavelength (nm)^b^	1443
Laser energy density (mJ/mm^2^)^b^	49
Structural parameters^a^	
*R_g_* (Å)	18.1 ± 0.07^c^
	17.8 ± 0.1^d^
*I*_0_ (arb.)	0.09 ± 3 × 10^–4^^c^
	$0.09\pm 7\times 10^{-4}$^d^
*D_max_* (Å)	56.2^d^
MW (kDa)	16.8^e^
	16.9^f^
Data processing	
Radial integration	Own Python code
Data processing	ATSAS 3.0.1^a^
	Own MATLAB code^b^
Structural modeling	
Computation of intensities	CRYSOL (ATSAS 3.0.1)
MD simulations	GROMACS 5.0.4
	PLUMED 2.5.1
MD starting structure	PDB ID: 1BVC, random coil
Structural refinement	EOM, adapted
*q*-range used (Å^−1^)	0.03–0.3

^a^ Conventional SAXS.

^b^ TRXSS.

^c^ Results obtained from the Guinier analysis.

^d^ Results obtained from the pair distribution function [P(r)].

^e^ The molecular weight (MW) was estimated from the sequence.

^f^ The MW was estimated from the MW assessment tool.^69^

We induced a T-jump of 10 °C, with a 7 ns infrared laser pulse, in the sample, which perturbed the equilibrium in favor of the unfolded state at 60 °C (Fig. 3). TRXSS was recorded from 10 ns to 600 *μ*s after the T-jump to monitor the unfolding process of apoMb [Fig. 5(b)]. The heating signal in the data was subtracted as described in Material and Methods (Fig. S2).

The difference scattering signal evolved over time, reflecting the structural changes of the protein. Within the first 60 ns, a negative difference signal is observed at *q* < 0.15 Å^−1^ [Fig. 5(c)]. This part of the difference scattering curve is mostly influenced by changes in long distances and, thus, the global shape of the solute.^70^ The signal at low *q* and early times has been observed by others^53^ and is associated with rearrangement of the solvation shell and thermal expansion of the protein. From 1 *μ*s, a depression appeared at q≈ 0.11 Å^−1^ and increased in amplitude until 600 *μ*s, suggesting conformational changes on the scale of the entire protein [Fig. 5(c)]. After ca. 100 *μ*s, oscillations in the difference scattering curve appeared (0.2 < *q* < 0.6 Å^−1^). Changes in this *q* range are related to changes in the secondary and tertiary structures.^33–35^

A singular value decomposition (SVD) was performed on the TRXSS data (Fig. S4). Even though we did not use the SVD decomposition in further analysis, this operation provides an unbiased estimate of the number of components required to explain the data. Based on the eigenvalues of the SVD, at least three species are expected in the data [Fig. S4(b)].

### Refining ensembles against the scattering data

It is common to subject time-resolved data to an analysis, where the transition from one transient structure to another is assumed to follow a predefined kinetic model.^35,48,53,71^ This approach works well for transitions that are essentially unidirectional. However, for folding equilibria where the forward and reverse transitions occur with similar rates, this analysis becomes more complicated. At every given time point, a mixture of protein states will be present and the pathway, which connects the states, is not known *a priori*. To avoid any of the presuppositions, which are associated with kinetic models, we fitted ensembles of protein structures to the SAXS data recorded at 50 °C and to the difference scattering signals at each time point.

For this, we used an ensemble optimization method (EOM). The EOM was originally developed in the ATSAS package to refine ensembles of structures against SAXS data and has been used for disordered proteins^49,72^ and unfolded proteins.^73^ Here, we further extend the method for difference scattering used in TRXSS to refine ensembles of structural changes. In our implementation, a set of ca. 16400 candidate structures were generated. To ensure that these structures cover a wide conformational space from folded to unfolded apoMb (Fig. S3), we used three sets of MD simulations. Two sets of simulations started from the crystal structure of myoglobin [Protein Data Bank (PDB) accession code: 1BVC] after the removal of the biliverdin ligand [Fig. 1(a)]. The protein was simulated either at elevated temperature (from 300 K to 520 K in 20 K steps) or by moving *R_g_* restraints with PLUMED.^62,63^ A third set was obtained by starting the simulations from a random coil model of apoMb. After generation of the trial models, their theoretical scattering was computed with CRYSOL.^44^ Ensembles of structures were refined against difference data using an iterative genetic algorithm (see Materials and Methods for details). For each set of data (SAXS at 50 °C and each TRXSS time point), the genetic algorithm was repeated 100 times, resulting in 100 ensembles, each consisting of 50 structures. The best scoring quartile of these ensembles was analyzed further.

The scattering of the ensembles, refined against the 50 °C SAXS data, shows excellent agreement over the entire *q* range [Fig. 5(a)]. The ensembles for the TRXSS difference data were selected with respect to the average refined scattering of the 50 °C data. Figure 5(b) presents the scattering of the ensembles refined against the different time points of the TRXSS data. We observed some discrepancies between the fits and the experimental scattering at low *q* and delay times longer than 100 *μ*s. The amplitude of the experimental scattering is consistently higher than that of the fitted scattering. We see three possible explanations to this discrepancy. The first is that the pool of trial structures does not contain suitable candidates. The second is an inappropriate approximation of the hydration layer by CRYSOL as previously reported for disordered proteins.^64,74^ The third is that we observe the onset of a temperature dependent structure factor with increased attractive interaction between protein molecules. This discrepancy is also visible when assessing the refinement scale factor and the ensemble average hydration layer contrast (Fig. S5). These values remain relatively constant until ca. 30 *μ*s when they both start to increase. Because the change in the scale and hydration layer largely coincides with the deterioration of the fits (after ca. 100 *μ*s), we are unable to draw any conclusions regarding this.

**FIG. 6.**
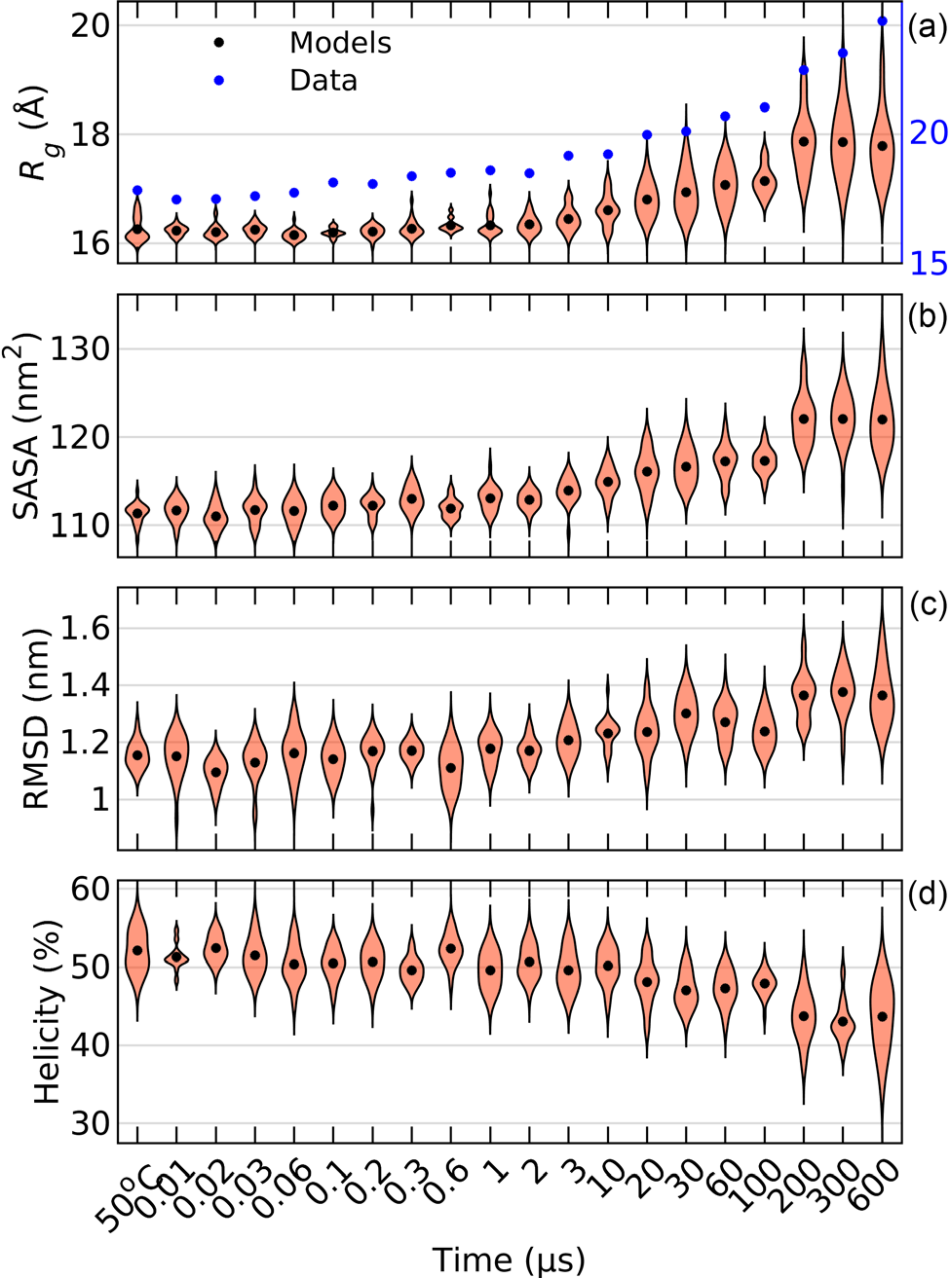
Density distribution for *R_g_* for models (red) and estimated directly from data (blue) (a), SASA (b), RMSD (c), and helicity (d) for the ensembles selected by the genetic algorithm for the 50 °C SAXS data and for the TRXSS data at each time point. The average in each distribution is represented with a black dot.

To structurally characterize the unfolding process of apoMb, we calculated the radius of gyration (*R_g_*), the solvent accessible surface area (SASA), the root mean square deviation (RMSD) with respect to the crystal structure, and the helicity of each atomic model selected by the genetic algorithm (Fig. 6). In addition to the model derived *Rg*, the Guinier approximation was used to calculate the change in *R_g_* directly from the data, using difference scattering for q≤ 0.05 Å^−1^ [blue dots in Fig. 6(a)]. This shows how the average structure changes over time. An increase in *R_g_* as well as SASA and RMSD is observed, along with a decrease in helicity. This suggests that the protein loses its structure. In addition, the distribution of *R_g_*, SASA, RMSD, and helicity also increases at later time points, indicating increased flexibility. The *R_g_* of the atomic models and that calculated directly from the data follow each other relatively well (correlation coefficient of 0.98) but start to diverge at longer time points. The *R_g_* derived from the atomic models is consistently lower than that suggested by the data. This is because the *R_g_* that is calculated from the simulations represents the protein *in vacuo*, whereas the experimentally determined *R_g_* also includes the hydration layer (see Table S1 and Ref. 75).

### Cluster analysis identifies three structural groups in the apoMb unfolding pathway

**FIG. 7.**
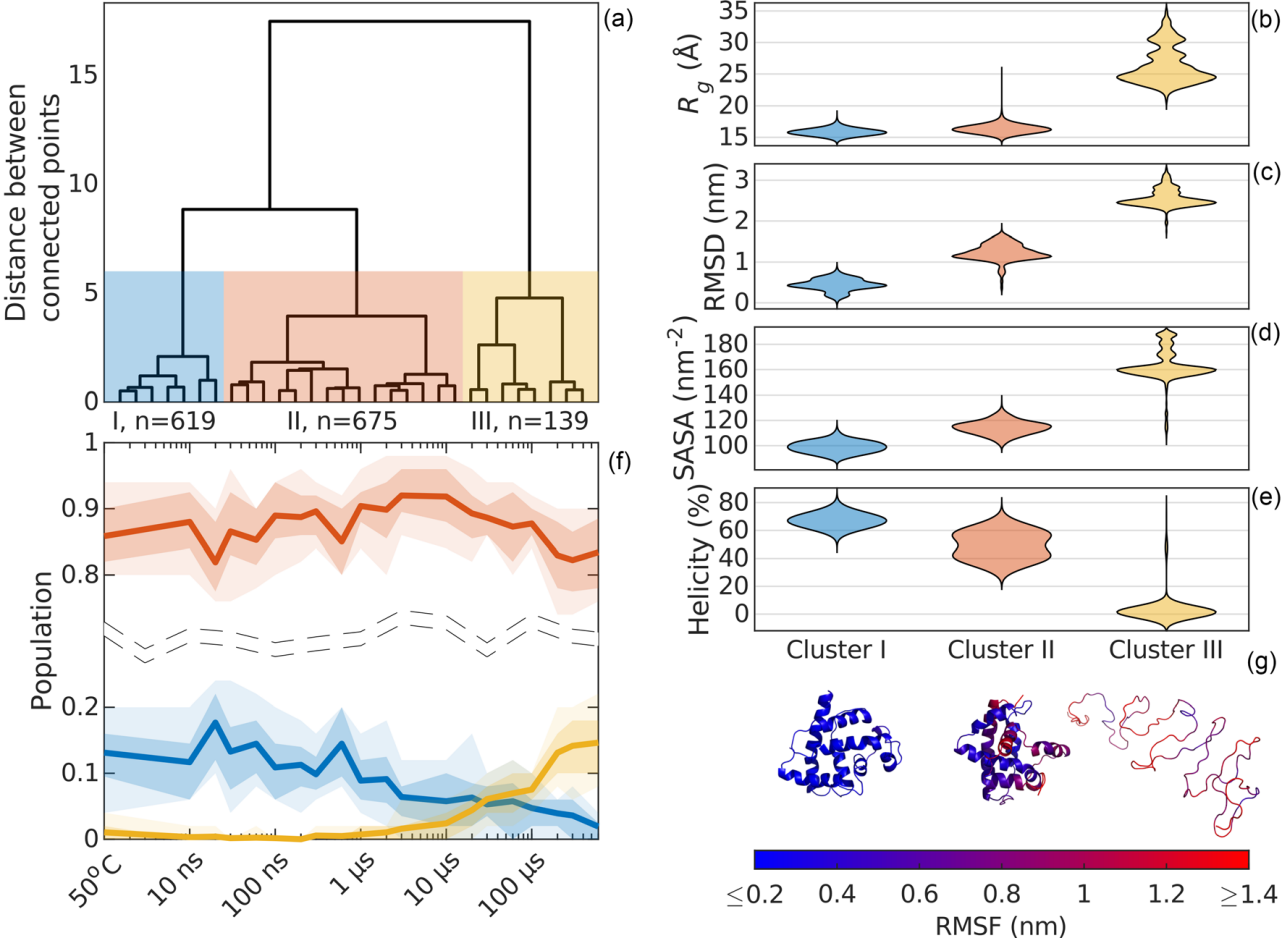
Cluster analysis. Hierarchical clustering of the structures according to their *R_g_*, RMSD, SASA, and helicity reveals three clusters at a cutoff of six normalized distance units (a). The distribution of *R_g_* (b), RMSD (c), SASA (d), and helicity (e) for the three clusters. The population of each cluster at the different time points (f). The light shaded area represents the 10th to 90th percentile and the darker shaded area the 25th to 75th percentile. The most frequent members of each cluster are depicted in panel (g), where the coloring indicates the root mean square fluctuation (RMSF) within each cluster.

The structures selected by the genetic algorithm were clustered according to their *R_g_*, SASA, RMSD, and helicity [Figs. 7(a)–7(e)]. Depending on the selected cutoff, a varying number of clusters will emerge. The SVD analysis suggested that three species are required to explain the data, and the cutoff was set accordingly. We identified three clusters: I–III [Fig. 7(a)], with 619, 675, and 139 unique structures, respectively. The relative abundance of structures belonging to the different clusters over time was calculated [Fig. 7(f)]. This revealed that structures belonging to cluster II dominate the ensembles (ca. 80%–90%) at all time points. Structures belonging to cluster I are found at early time points (ca. 15%) and decrease in frequency after ca. 1 *μ*s; at the same time, we see that the contribution of structures from cluster III increases to a maximum (ca. 15%) at 600 *μ*s.

### Structural analysis of the clusters

**FIG. 8.**
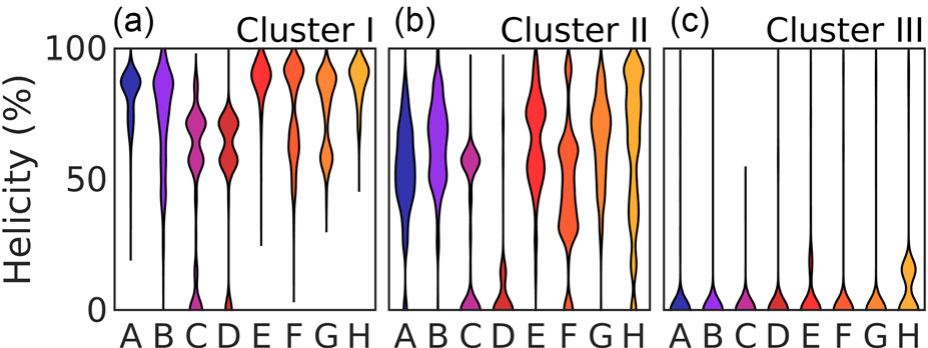
Distribution of the helicity of the eight *α*-helices (a)–(h) in the different clusters.

**FIG. 9.**
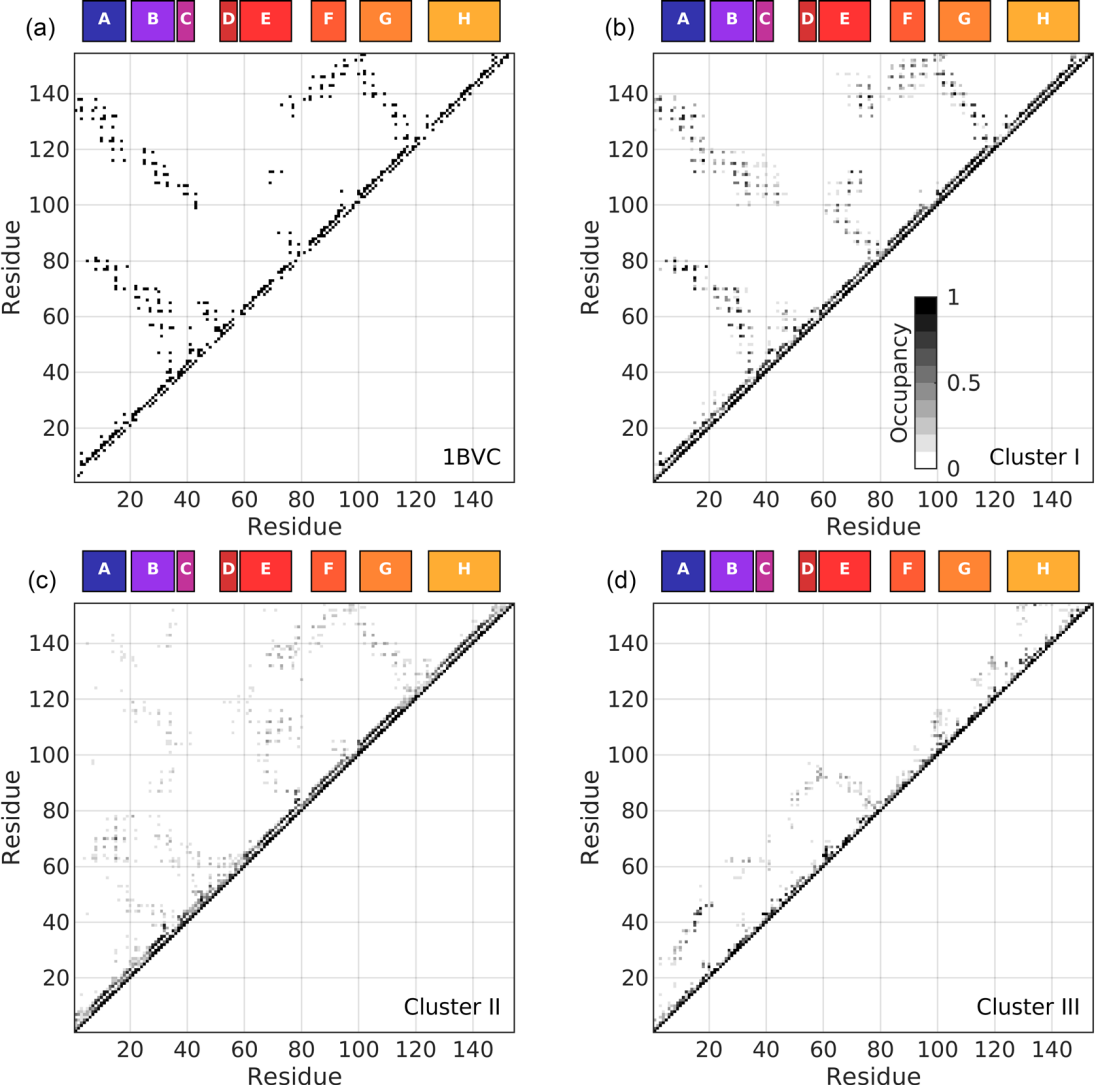
Contact maps for the crystal structure (1BVC), after removing the biliverdin ligand and adding hydrogen atoms (a) and three clusters I (b), II (c), and III (d). Residue pairs closer than 4 Å are considered to be in contact.

The atomic models comprising the three clusters I–III were analyzed in terms of *R_g_*, SASA, and RMSD with respect to the crystal structure and their helical content (Table II, Fig. 8). We also assessed the contacts between residues (Fig. 9). The side-chains of two residues were considered to be in contact when they were located within 4 Å of each other. The *R_g_*, SASA, RMSD, helical content, and number of contacts per cluster are the weighted averages of each cluster. This means that structures selected more times by the genetic algorithm have a larger influence on the cluster average than those selected fewer times.

**TABLE II.** Structural parameters for the different clusters.

	Cluster I	Cluster II	Cluster III
*R_g_* (Å)	15.83 ± 0.36	16.32 ± 0.55	26.42 ± 2.88
SASA (nm)	98.93 ± 4.48	114.27 ± 5.24	162.85 ± 12.88
Helicity (%)	67.13 ± 3.99	49.24 ± 9.68	2.95 ± 8.36
RMSD (nm)	0.44 ± 0.14	1.23 ± 0.21	2.56 ± 0.22

Molecular models belonging to cluster I were found in the steady-state SAXS and the TRXSS data. At delay times shorter than 1 *μ*s, cluster I represents about 15% of the structures, after which their population decreased. Individual inspection of a subset of the cluster members suggested that cluster I represented folded proteins, and there is little flexibility as indicated by the intra-cluster RMSF [Fig. 7(g)]. The average *R_g_* of this cluster was ca. 16 Å (Table II), which correlated well with the previously published results for MD simulations of apoMb.^76^ As mentioned previously, compared to SAXS, *R_g_* calculated from MD simulations is typically smaller on the order of 2 Å (see Table S1 and Ref. 75). Considering this, the calculated *R_g_* of ca. 16 Å is also in agreement with the *R_g_* of ca. 18 Å observed with SAXS (Table I and Refs. 37 and 68). The *α*-helices of apoMb were mostly folded [Fig. 8(a)]. The average total helicity was about 67% (Table II), also similar to the previously observed helicity for apoMb.^17^ The RMSD compared to the starting crystal structure was relatively low. Cluster I also displayed the lowest SASA of all clusters, indicating a compact structure where residues are protected from the solvent (Table II). In addition to the contacts between residues *i* and *i* + 4 forming a turn of an *α*-helix, cluster I showed that many inter-residue contacts between helices are also found in the crystal structure [Figs. 9(a) and 9(b)]. Native contacts (contacts present in the crystal structure) were observed between helix A and helix B and helices E, G, and H. Similar contacts have previously been found in folded apoMb.^76^ The structural parameters derived from the TRXSS analysis indicate that cluster I contains structures resembling the compact and folded apoMb, which we will refer to as native.

Cluster II was highly populated in all time points [Fig. 7(f)]. The structures found in this cluster generally appeared to be less folded than in cluster I [Figs. 7(b)–7(e)] and more variation was seen within this cluster [Fig. 7(g)]. The *R_g_* and the SASA are also larger than in cluster I (ca. 3% and 16%, respectively, Table II). Expanded *R_g_* for apoMb unfolding intermediates has previously been reported by MD^76^ and conventional SAXS.^37^ We observed a decrease in the helical content of the *α*-helices in cluster II compared to cluster I, with only 49% left, mostly attributed to loss of helicity in helices C, D, and F [Fig. 8(b)]. We also noted a wider distribution of the helical content in our models for cluster II compared to cluster I. This was particularly prominent for the short helices (six residues) C and D. Also, cluster II shows a pattern of inter-residue contacts that shares several similarities to the crystal structure and native state. However, the contacts appear to be of a more transient character, with occupancies lower than what is observed in cluster I [Fig. 9(c)]. Still, the most commonly observed contacts are contacts between helices A, E, G, and H, similar to the crystal structure. Based on these observations, we defined cluster II as expanded, partially folded, and with a compact hydrophobic core. This intermediate shows several qualitative similarities to the molten globule intermediates previously found through acid and urea equilibrium denaturation experiments.^10,16,37,68,77^ However, the intermediate identified here appears closer to the native structure.

Structures belonging to cluster III started to appear from 1 *μ*s, increasing to represent ca. 15% of the population at 600 *μ*s [Fig. 7(f)]. This cluster was largely devoid of structure [Fig. 7(g)] and characterized by a large *R_g_* (ca. 67% larger than cluster I) and an expanded SASA (ca. 65% increase), indicating pronounced solvent penetration (Table II). A similar value for the *R_g_* was found for the acid unfolded apoMb in MD simulations.^76^ The helical content was less than 3%, with only a small part of helix H being folded [Fig. 8(c)]. In cluster III, all long range contacts are missing [Fig. 9(d)]. Expanded *R_g_* and decreased helicity are also seen in the acid unfolded apoMb at pH around 2.^9,17,76^

## DISCUSSION

### Different approaches to study protein (un)folding

Before discussing the results of the present study in greater detail, it may be worth briefly considering some differences between various approaches used to studying protein (un)folding, in terms of what it is that is observed. Frequently, folding and unfolding of proteins are considered to be similar pathways but in opposite directions. However, (un)folding experiments are typically performed under conditions that favor either the folded or some form of unfolded state of the protein. Depending on how this shift toward either conformation is achieved, it is not certain that folding and unfolding experiments actually do reveal similar pathways. For example, the experimental conditions may strongly favor one of the states and greatly change the topology of the free energy landscape, essentially making the reaction unidirectional.^78^ In our experiments, we study the conformational changes in apoMb at near equilibrium conditions, shifting the population of protein conformations through a temperature increase. In such an experiment, the folding and unfolding pathways are the same, and only the equilibrium population is shifted. In contrast, in a recent study by Kim *et al.*,^79^ the authors trigger the folding of cytochrome c via a light induced electron injection and monitor the folding by TRXSS. This places the protein far from equilibrium and alters the energy landscape. What they observe is an essentially unidirectional reaction. Compared to the reversible reaction triggered by modest temperature jumps, such light triggered conformational changes may not reveal similar folding and unfolding pathways.^80^

Several techniques have been used to investigate the (un)folding of proteins.^9,10,17,37,77,81,82^ Methods that are directly sensitive to structural changes are scarce. While some established techniques, such as NMR or SAXS, readily provide structural information of ensembles at equilibrium, accessing transient species is more difficult. These equilibrium experiments require the presence of stable intermediates in protein unfolding pathways.^37,68,83^ This limits the versatility and applicability of those methods to a restricted fraction of proteins and intermediates. It is also not always clear if the stabilized state corresponds to the intermediates that occur in the folding reaction. To fully understand the unfolding process of proteins and their biological significance, kinetics and time-resolved studies are essential.

TRXSS is a direct structural probe. It is sensitive to both small and large-scale conformational changes and is able to detect such changes with a high time-resolution. We have recorded TRXSS data on a nano- to millisecond timescale to observe the different conformational states in the apoMb unfolding pathway. During the course of a dynamic experiment, populations of the different structural states are constantly changing. It is, thus, also very important to disentangle the conformational heterogeneity of the protein population. Instead of only analyzing the protein solution as a homogeneous entity (e.g., Refs. 15, 82, and 84), we fitted ensembles of structures to the data. As such, we implement a new strategy for the interpretation of TRXSS data.

A challenge in analyzing x-ray solution scattering data lies in the prediction of scattering form atomic models. Currently available methods have their weakness either in their coarseness (e.g., CRYSOL) or in their high computational demand (e.g., WAXSiS). Their high computational demand makes the more accurate methods useful only for validation and not for exploring a wide range of possible conformations. Exploratory strategies, such as the one described herein, would greatly benefit from improved tools for predicting x-ray solution scattering from atomic coordinates in an accurate yet efficient way.

### Sequence of apoMb thermal unfolding

**FIG. 10.**
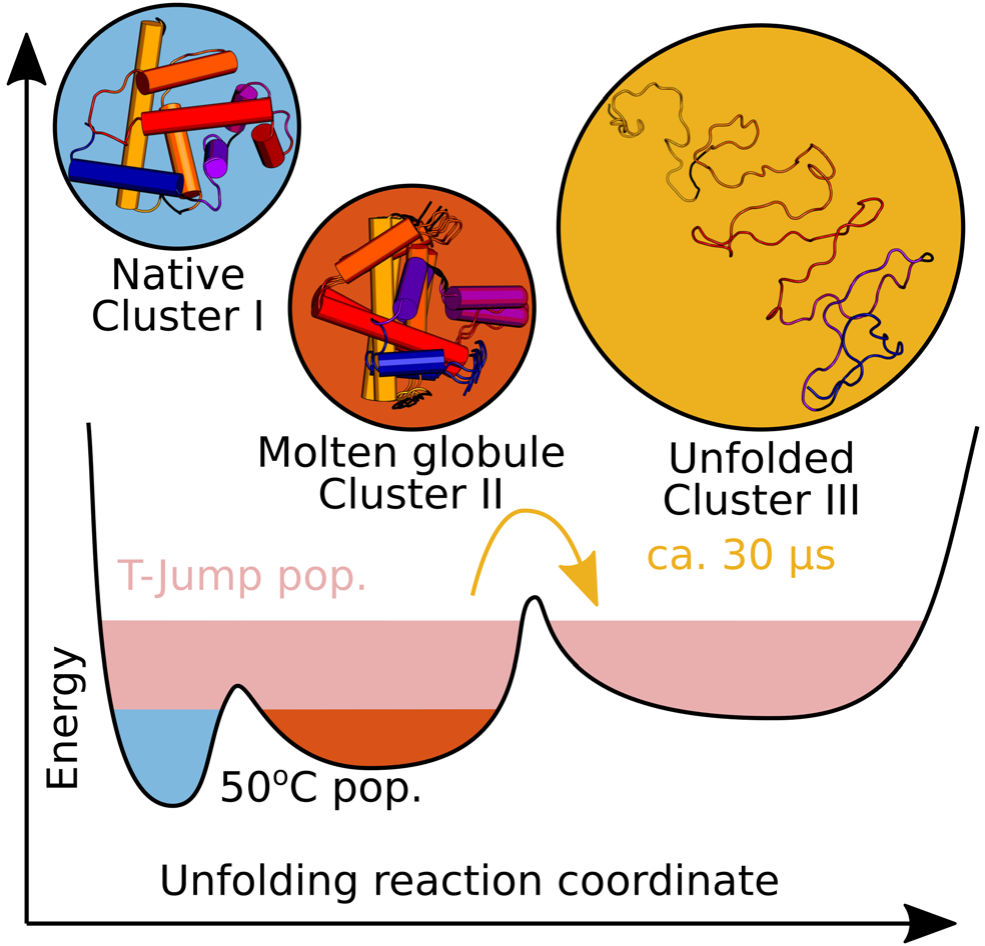
At 50 °C, both native and molten globule-like structures are found in the ensemble. The T-jump alters the equilibrium populations, favoring unfolded apoMb. Unfolded structures start to appear ca. 1 *μ*s following the T-jump and are present in larger amounts than the native structures after ca. 30 *μ*s.

Studies of apoMb (un)folding under chemical equilibrium have revealed three macroscopic states present during the unfolding of the protein: the native, the molten globule, and the unfolded state.^9,10,17,37,77,85,86^ Our data show that these conformational states indeed occur in the unfolding process within the first 600 *μ*s as the protein ensemble adjusts to a 10 °C T-jump (Fig. 10). Based on the structural analysis of the clusters I, II, and III and their time-dependent concentration, we are able to order the events that lead to the unfolding of apoMb and directly probe the formation of transient states in real-time.

At our starting temperature of 50 °C, and for the first microseconds following the T-jump, cluster I represents ca. 15% of the population. This cluster resembles the native protein, with a *R_g_*, and helicity close to what has been found previously.^17,37,68^ It possesses a small RMSD with respect to the myoglobin crystal structure. At a first glance, this is not in agreement with the folded/unfolded fraction derived from tryptophan fluorescence (Fig. 3) and from CD^83^ or IR^51^ measurements. However, if the helicity and abundance of cluster I and II are considered, about 80% of the secondary structure (compared to cluster I) is still retained. Additionally, the tryptophan residues in apoMb are located on helix A, which is largely folded even in cluster II.

At all time points investigated, as well as the equilibrium measurement at 50 °C, structures belonging to cluster II dominate the ensemble. The atomic models have a preserved hydrophobic core and maintain an overall folded structure (Table II). Compared to the native state, helices C and D [Fig. 8(b)] of this intermediate are unfolded, and helix F shows a distinct decrease in helicity, resulting in an overall decrease in the helical content (Table II) and a larger *R_g_*. This intermediate qualifies as a molten globule.

Approximately 1 *μ*s after the T-jump, unfolded structures start to appear in the ensemble (cluster III), and after 30 *μ*s, they outnumber the folded structures. The unfolded state shows substantial deviations from the native (cluster I) and the molten globule state (cluster II). Structural analysis of the members of this cluster reveals that they are characterized by an expanded *R_g_* and a drastic increase in RMSD and SASA (Table II). We also detect an almost complete loss of helicity [Fig. 8(c)].

### Equilibrium vs kinetic intermediates

The native state identified in this study closely resembles native structures observed by others.^37,68,76^ The partially folded intermediate, represented by cluster II, qualitatively resembles the molten globule intermediates found under equilibrium conditions at low pH.^10,16,17,37,68,77^ In agreement with recent unfolding studies, performed by quench-flow hydrogen exchange and CD,^87,88^ we see that helix E docked on helix A is substantially folded and stable in the molten globule.

However, when comparing our “kinetic molten globule” to the previously identified “equilibrium molten globules,” we find quantitative differences. In earlier studies, the equilibrium molten globule had an expanded *R_g_* compared to the native state^37^ of ca. 20%. In contrast, the kinetic intermediate only shows ca. 3% expansion. The helicity (ca. 49%) of the kinetic intermediate is similar compared to the low pH molten globule (ca. 45%).^17^ Most of the molten globule observations were described at low pH and equilibrium. The difference observed here might indicate a somewhat different unfolding pathway under non-equilibrium conditions. Different conditions are likely to result in different protein structures.

It cannot be excluded that the previous description of the molten globule obtained at equilibrium presented an average for a mixed population containing also a fraction of native as well as unfolded structures (Fig. 10). By means of TRXSS and ensemble analysis, we extracted information that pertains to the molten globule alone rather than the average of the population. Dominating the ensemble at 50 °C and during the first 600 *μ*s after a 10 °C T-jump, it possesses a smaller *R_g_* but only a slightly higher helicity than previously reported for the equilibrium molten globule. The analysis time-resolved scattering data confirm and extend prior findings that the structure of the apoMb kinetic molten globule is rather similar to that of the native protein.^87^ In fact, a similar overall structure may make the molten globule intermediate and the native protein difficult to distinguish from each other.

The unfolded structures found in cluster III have a lower *R_g_* than what has been observed before for unfolded apoMb.^15,17,37,68^ However, there is also substantial variation in the previously published *R_g_* of unfolded apoMb. This is likely because there is no defined structure and slight variations in experimental conditions will promote more or less expanded states. It should be noted that for unfolded structures, the scattering profiles computed using CRYSOL are expected to deviate the most from the real scattering,^64^ and that it is at time points when the unfolded structures are found that we have the least agreement between the experimental and modeled data. It should also be noted that the complete loss of helicity that we see in cluster III may be an artificial observation, based on the inability to sample conformations with a helicity of ca. 10%–30% (Fig. S3). The results are qualitatively in line with what Dametto and Cárdenas^89^ observed in MD simulations of sperm whale apoMb folding at 300 K, which is a sharp decrease in helicity for *R_g_* above 16 Å. However, they typically observe ca. 13% retained helicity even for large *R_g_* and an unfolded protein. Nevertheless, the conclusion presented here that cluster III represents unfolded apoMb and is largely devoid of a defined three-dimensional structure can still be expected to hold.

### Contacts involved in the upkeep of apoMb structure

The MD simulations, and the resulting structures underlying the analysis, provide precise information about the position of each atom. These coordinates can be used to assess which residues are in contact. Indeed, for the structures in cluster I, a large number of native inter-residue contacts are found, long range (e.g., helices A–H) as well as short (e.g., helices B and C) and medium range (e.g., helices E and F or E–G). As the protein starts to unfold (cluster II) and side-chains get exposed to solvent, the occupancy and number of native inter-residue contacts start to decrease. In the molten globule intermediate (cluster II), most native contacts decrease in occupancy and non-native contacts start to appear. We note that the contact pattern observed in the crystal structure becomes increasingly diffuse in going from cluster I to II, reflecting an increased structural flexibility. This is also reflected by the more than double number of contacts (occupancy aside) found in the molten globule intermediate (cluster II) compared to the native state (cluster I). The hydrophobic core, composed of helices A, G, and H (AGH core), is still maintained by inter-residue contacts; otherwise, most contacts are in the close or medium range. We note that the network of inter-residue contacts is particularly well conserved for helices E–H going from the crystal to the native (cluster I) to the molten globule (cluster II).

## CONCLUSION

Studying the conformational transitions during protein folding in real-time as the sample adapts to a new thermal equilibrium is a grand challenge. In this contribution, we show that TRXSS, coupled with a laser induced T-jump, is capable of detecting unfolding as it is happening. We also detail a new strategy for the structural analysis of the TRXSS data using MD simulations and ensemble optimization methods. The analysis yields information on how the global structure of apoMb changes during unfolding. ApoMb is already in a mixture between native and molten globular states at 50 °C before the T-jump is introduced (Fig. 10). After the T-jump, unfolded conformations start to appear after ca. 1*μ*s and increase in number over the following 600 *μ*s (at least). Using the atomic models generated by MD and filtered using TRXSS data, insights are provided into the secondary structure and inter-residue contacts, which are important for the three-dimensional structure of this protein. Together, these tools provide a powerful basis for further investigations of protein folding.

## SUPPLEMENTARY MATERIAL

See the supplementary material for additional figures and information on simulations of the laser induced temperature jump.

## AUTHORS' CONTRIBUTIONS

O.B., S.W., and M.R.P. designed the research and planned the experiments. L.H., M.R.P, L.I., E.C., I.K., R.H., S.W., and O.B. performed the experiments. O.B. and L.H. analyzed the data. L.H., S.W., and O.B. wrote the manuscript with input from all others.

## Data Availability

Raw data were generated at the BioCARS beamline (ID14-B) at the Advanced Photon Source (APS) large scale facility. The derived data that support the findings of this study are openly available in Figshare at https://doi.org/10.6084/m9.figshare.12011844, Ref. 90.
